# A feasibility study of the impact of a communication-skills course, ‘Empowered Conversations’, for care partners of people living with dementia

**DOI:** 10.1177/14713012211018929

**Published:** 2021-05-17

**Authors:** Lydia Morris, Anthea Innes, Emma Smith, Tracey Williamson, Phil McEvoy

**Affiliations:** Institute of Dementia, School of Health and Society, 7046 University of Salford, UK; Division of Population Health, Health Services Research and Primary Care, University of Manchester, UK; Institute of Dementia, School of Health and Society, 13995 University of Salford, UK; Six Degrees Social Enterprise, Salford, UK; Association for Dementia Studies, 8709 University of Worcester, UK; Six Degrees Social Enterprise, Salford, UK; Age UK Salford, Eccles, Manchester, UK

**Keywords:** dementia, caregivers, carers, training, psychosocial interventions

## Abstract

**Objectives:**

To examine the feasibility, acceptability and impact of an experiential course for family care partners of people living with dementia, Empowered Conversations (EC). This study aimed to assess the impact of participation in an EC course on care partner stress levels, communication and mentalization (a form of relational-based empathy).

**Method:**

This study uses an uncontrolled pre–post-follow-up design. Follow-up was at 4-months after the initial EC session where baseline data were collected. One hundred and fifty-nine care partners were recruited. EC is a training course that has been designed to improve care partner communication, well-being and relationships. It is based on an integrative framework that targets the specific psychological, relationship and communication needs of carers. This framework informs targeted strategies and interactive exercises that facilitate carers to consider the goals and emotions of those they are caring for, alongside their own goals and emotions, and to use this to maximize good communication.

**Results:**

Stress was significantly reduced across the three time points. Communication significantly improved across time. There were no significant changes in reflective functioning (mentalization).

**Discussion:**

This study provides preliminary evidence that a communication-skills training course for care partners of people living with dementia is an acceptable and feasible intervention and has an impact both post-intervention and at follow-up. These findings require validation in a rigorous, randomized study.

Family carers provide intense practical support, whilst coming to terms with changes in their relationship (Morris et al., 2018a). Research indicates that about 40% of family carers^1^ of people with dementia have clinically significant depression or anxiety (Cooper et al., 2007). Challenges to communication present a significant source of frustration, low mood and stress for both people living with dementia and their carers (Dooley et al., 2015; Holst & Hallberg, 2003; Steeman et al., 2006), but improved communication can enhance relationships and support people living with dementia to feel connected and understood (Judge et al., 2013; La Fontaine & Oyebode, 2014). A meta-synthesis and a systematic review have both highlighted how the quality of the relationship with the primary care partner impacts on well-being and the experience of dementia for both parties (Ablitt et al., 2009; Wadham et al., 2016).

Two systematic reviews of communication-based training interventions for carers of people living with dementia have identified limitations to the interventions available (Eggenberger et al., 2013; Morris et al., 2017). A minority of interventions demonstrated improvements in care partner anxiety and depression (e.g. Gitlin et al., 2010; Judge et al., 2010; Livingston et al., 2013). High levels of anxiety and depression are likely to impact on care partner communication. In spite of this, courses targeting communication tend to have a narrow focus on the pragmatics of communication; for example, delivering limited content information in short, simple sentences (Eggenberger et al., 2013; Morris et al., 2017). While such strategies can be helpful, they do not encompass changing relational dynamics; for example, a spouse or child may feel increasingly cast in a parent role (Pozzebon et al., 2016; Smebye & Kirkevold, 2013). Very few interventions for family care partners of people living with dementia demonstrate improvements on specific communication outcomes, and even when effects were demonstrated these were not consistent across follow-up points (Morris et al., 2017). Generally, these interventions have a limited effect on care partner quality of life (Eggenberger et al., 2013; Morris et al., 2017).

The ‘Empowered Conversations (EC)’ course was developed in response to this need (Morris et al., 2018a). EC is a group-training course for care partners of people living with dementia that has been designed to improve communication, well-being and relationships. It is a weekly training course delivered over four consecutive weeks (2.5 hours per week, including a break) at accessible community venues.

EC is based on the Communication Empowerment Framework, which integrates evidence-based models to address the specific psychological, relationship and communication needs of carers (Morris et al., 2018a). This innovative theoretical framework builds on person-centred principles but delineates in detail the communication and relational components that can contribute to the well-being of care partners and people living with dementia. The Communication Empowerment Framework draws together three theoretical accounts of how humans negotiate their world and maintain well-being: (1) Mentalization Theory relates to our perceptions of ourselves and others; (2) Perceptual Control Theory explains how conflicting internal and interpersonal goals can reduce control and impact on our relationships and (3) The Communicative Impact Model accounts for the complex and contradictory challenges of dementia care in terms of the intricate parameters of successful communication. These three models and their integration are described in detail in Morris et al. (2018a). The synthesis of these three accounts informs targeted strategies and interactive exercises that facilitate carers to consider the goals and emotions of those they are caring for, alongside their own goals and emotions, and to use this to maximize good communication.

The Communication Empowerment Framework specifies how both internal and external conflicts can arise. An example of an internal attachment conflict that family care partners may face is the conflict between wanting the person to be ‘as they were’, yet having to deal with them ‘as they are’, that is, with the cognitive, memory, communication and personality changes resulting from dementia. Good ‘mentalizing’ by the care partner is important in maintaining a positive relationship with the person they are caring for (Jain et al., 2014; McEvoy et al., 2019). Good mentalizing is defined as: ‘an accurate and effective understanding of (a) his or her personally important goals; and (b) the other person’s perspective that takes into account what is really important to them (Morris et al., 2018a, p. 7).A key focus of the Framework is recognizing how communicative behaviours on the part of the care partner can unintentionally undermine the experience of control in the person that they are caring for (Morris et al., 2018a). For example, if the care partner is aggressive, or unable to achieve basic communicative goals because of an internal conflict, the person living with dementia will have a reduced range of options for responding (such as, if the care partner is unable to adapt their communication to changes that the person they are supporting is experiencing because they are conflict about treating the person ‘as they are’, instead of ‘as they were’). Since enduring goal conflicts are likely to cause distress and stress (Gray et al., 2017; Kelly et al., 2015), helping care partners to work around them is crucial. In order to reduce undermining behaviours by the care partner, carers need to be supported in effective mentalizing that takes into account the current situation and does not remaining in thrall to historical goals that can no longer apply. Care partners are taught practical ways to increase control for the person living with dementia, such as the ‘invitation to respond’ technique, which allows people living with dementia to respond if they want to (Morris et al., 2018a).

The aim of this study is to report on the impact of the EC course on care partners, on their stress levels, communication, mentalization and understanding and satisfaction with EC. In line with Medical Research Council guidelines, this feasibility study collects data that can improve the quality and impact of the intervention, manual and training process (Craig et al., 2008). Further, secondary aims were to examine the acceptability and feasibility of EC. Whether EC was acceptable to care partners was primarily examined using satisfaction and understanding ratings. Feasibility of future delivery and research was assessed based on recruitment, retention and utility of intervention materials (e.g. Cramer et al., 2011; Sumathipala et al., 2000).

## Methods

### Participants

One hundred and fifty-nine care partners were recruited from a range of third-sector organizations across Greater Manchester, a densely populated, predominantly urban geographical location in England. Participants were eligible if they were informally caring for someone living with dementia, wanted to attend a training course and were able to give informed consent. They needed to have sufficient English language skills to understand the training (i.e. verbal and written language abilities required to understand verbal presentations and complete simple exercises). There were no specific exclusions regarding psychological health, but the research team included both a Clinical Psychologist and a Mental Health Nurse who developed risk management and signposting procedures. The facilitators were also trained in these procedures. It was not necessary to exclude anyone from the study on the basis of psychological health, but if there were concerns regarding a care partner’s well-being, the research team ensured that the care partner was accessing other support (e.g. personal psychological therapy). For example, all care partners completed a questionnaire relating to stress at the beginning of their first EC session, and if stress levels were very high, the researcher would ask the care partner questions about this and provide signposting information if required.

### Measures

Primary outcome measures were

*Perceived Stress Scale (PSS-10;* Cohen et al., 1983): The 10-item PSS was used. The PSS has been used previously with care partners (e.g. Cristancho-Lacroix et al., 2015). Higher scores represent higher stress levels.

*Carer Communication Questionnaire (CCQ)*: A 14-item questionnaire developed for the study. This questionnaire aimed to measure general communication understandings and skills. Example items include ‘I am aware of my body language when talking’ and ‘I am aware how my feelings or stress levels might impact a conversation’.

Process and confidence measures were

*Reflective functioning questionnaire (RFQ*; Fonagy et al., 2016): A 12-item questionnaire, adapted from the 8-item RFQ that primarily captures mentalizing in individuals with psychopathology. This measure captures tendencies towards ‘hypomentalization’, which describes an inability to consider complex models of one’s own mind and/or that of others. It also captures ‘hypermentalization, which describes excessive mentalizing, such as detailed accounts of another’s mental states without these being linked to observable reality. Four items were added to the RFQ to capture mentalizing within the relationship with the care partner more effectively. The items were adapted from 46- and 54-item versions of the RFQ. For example, ‘I believe there’s no point trying to guess what’s on someone else’s mind’, which was adapted to ‘I believe there’s no point trying to *guess what’s on the mind of the person I’m caring for’,* and ‘Other people’s thoughts and feelings are confusing to me’, which was adapted to ‘*The* thoughts and feelings *of the person I’m caring for* are confusing to me’.

*Satisfaction and evaluation measure*: This was created for this study and was completed anonymously; it was adapted from a satisfaction measure used in a previous prospective cohort study and RCT to evaluate the Take Control Course (Morris et al., 2016; Morris et al., 2018b). There were 2 versions: sessional and final session evaluation measure. The sessional evaluation measure included two 11-point scales: The first scale to rate satisfaction with the treatment and the second to rate understanding. Participants were prompted to provide written qualitative information regarding their experiences of each session, for example, ‘What have you found helpful about the session?’ On the final evaluation form, participants were asked additional questions, for example, ‘Have you noticed any effect on the people around you as a result of taking part in the course?’ These open-ended comments were used on an ongoing basis to make minor adjustments in the EC training protocol as the study proceeded (e.g. adding closed captioning to the films).

### Procedure

Recruitment was through third-sector organizations and carer networks, such as Age UK and local carer groups, who were visited at community group meetings and told about the study or passed the study information sheet by the project team. Posters and press were used to promote EC, and any care partners who enquired were sent the relevant study participant information sheet and arrangements made to follow this up if they were willing (having had a minimum of 24 hours to consider involvement).

Following written consent, pre-course measures were completed at the beginning of the first EC session. Post-course measures were completed at the end of the final session. Four-month follow-up measurements were generally completed over the phone; although occasionally, these were completed face to face.

The research was approved by the University of Salford Health and Society Ethics Committee prior to data collection (Ref. HSR1617-176).

### Intervention

Empowered conversations is a group-training course. It is based on an integrative framework that targets the specific psychological, relationship and communication needs of carers (Morris et al., 2018a). EC aims to create a space for care partners to pause, reflect and connect with peers. EC runs weekly for 4 weeks (each session lasts 2.5 hours and includes a break) and is delivered at accessible community venues. Throughout the course, care partners are encouraged to take a curious stance around communication. This stance helps participants who start the first session with a ‘x is doing this on purpose’ position and by the final session, this will have moved to ‘I wonder why x might be saying that?’ or ‘I wonder what x might be feeling?’ This approach encourages flexibility because care partners are given the space to identify and try out their own solutions using some of the techniques from the course.

#### Facilitators and intervention integrity

A step-by-step training process was used to train facilitators consistently. The initial stage consisted of attendance at two training sessions and observation of the experienced project manager facilitating a whole course. The training sessions covered key areas for good practice in delivering interventions in this context, such as confidentiality, group processes, risk management and safeguarding. Once the prospective facilitators felt confident, they delivered more of the content in a graded manner (gradually taking on more responsibility for leading more within the course) whilst supported by an experienced facilitator. EC materials are manualized, and the manual was a key training tool used with facilitators. Four facilitators were current or previous care partners of people living with dementia and had all initially attended the EC course as participants; the fifth had become interested in the course through their role as a healthcare professional but later became a carer for a family member. The facilitators were paid on a consultancy basis and came from a range of professional backgrounds (including GP, physiotherapist and fashion buyer), with some having substantial experience of delivering group education interventions.

Facilitators of EC received monthly external clinical supervision and weekly supervision from the project manager (who also facilitated courses).

The six facilitators each facilitated a range of courses (3–13). The mean number of courses facilitated was seven. The numbers of participants also varied across courses (3–19; mean = 6).

### Statistical analysis

Mixed-effects multilevel analyses were used to analyse data with a 2-level structure [responses at different time points (level 2) nested within individuals (level 1)]. The time points were baseline, post-intervention and 4 months after baseline. Mixed-effects models without any ad hoc imputation have been found to be more powerful than mixed-effects models with imputations for missing values (Chakraborty & Gu, 2009). Bootstrapping was used where outcomes were non-normal (Van der Leeden et al., 2008). We do not separately account for clustering at the group or facilitator level in this model, which is subsequently incorporated into the residual variation; our interest is in the estimates of the fixed effects. The models were estimated using maximum likelihood estimation, which includes all available data in the analysis. The xtmixed command in Stata 16 was used. Such models were used instead of repeated measures ANOVA because they include all available data (e.g. Chakraborty & Gu, 2009) and allow bootstrapping to account for non-normal outcome variables (e.g. Van der Leeden et al., 2008).

## Results

### Sample characteristics

See Table 1 for sample characteristics. Dementia diagnosis was reported by care partners, and a range of diagnoses were reported: Alzheimer’s disease 44 (26%); vascular 23 (13.6%) and Alzheimer’s disease and vascular dementia 8 (4.7%). Less common types of dementia were also represented, for example, frontotemporal and Lewy body dementia 6 (3.6%). However, not all care partners disclosed this (missing 41, 24.3%) and 26 (15.4%) specified ‘dementia’ rather than the specific type.

**Table 1.** Sample characteristics.

Variable	
Gender, no. female (%)	122 (72.2)
No. missing (%)	1 (0.6)
Age, M (SD)	59 (1.1)
Ethnicity, no. white (%)	143 (84.6)
No. Asian (%)	10 (5.9)
No. Black (%)	2 (1.2)
No. missing (%)	9 (5.3)
Non-spousal care partners (%)	98 (58)
No. missing (%)	15 (8.9)
Living with person with dementia (%)	74 (43.8)
No. missing (%)	86 (50.9)
Baseline PSS score, M (SD)	19.0 (6.8)
Baseline CCQ score, M (SD)	3.6 (0.5)

PSS: perceived stress scale; CCQ: carer communication questionnaire.

The EC courses were run across Greater Manchester, UK. The percentage of participants who attended courses across the boroughs are as follows: 20.7% in Salford; 19.5% in City of Manchester; 12.4% in Bury; 10.1% in Rochdale; 9.5% in Bolton; 8.9% in Oldham; 8.9% in Trafford; 5.9% in Wigan and 4.1% in Tameside.

### Retention

Two-hundred and forty-seven carers booked on the course. One hundred and fifty-nine attended the first session (20 were excluded; 35 cancelled and 27 did not attend), and 146 attended two or more sessions. Of those who cancelled, a range of reasons was given; for example, person caring for died, something else came up and could not attend time/location. Of those excluded, 85% were professional carers. Attrition levels from booking to attending were 36%, but once care partners attended, few dropped out.

### Outcomes

All 159 participants are included in the multilevel analyses. Multilevel analysis was conducted with stress and communication as the outcomes and time as the fixed effect term; it is detailed in Table 2. For the PSS, the main effect of time was significant, indicating that stress scores reduced over time. For the Carer Communication Questionnaire, the main effect of time was significant, indicating that communication scores improved over time. Table 3 reports the mean scores at each time point on all the measures.

**Table 2.** Multilevel analysis of the change of scores pre-intervention to follow-up.

	Overall model χ^2^(1)	Main effect of time		
		*b*	SE	95% CI
PSS	39.80***	−1.52***	0.24	−2.00, −1.05
CCQ	58.19***	0.17***	0.02	0.13, 0.21
RFQ (hypomentalizing)	0.16	0.01	0.02	−0.03, 0.04
RFQ (hypermentalizing)	0.00	0.00	0.02	−0.04, 0.05

Note: ***p < 0.001. PSS: perceived stress scale; CCQ: carer communication questionnaire; RFQu: reflective functioning questionnaire.

**Table 3.** Mean scores pre-intervention through to follow-up.

	Pre-intervention mean (SD)	Post-intervention mean (SD)	4-month follow-up mean (SD)
PSS	19.0 (6.8)	17.8 (6.6)	15.9 (6.1)
CCQ	3.6 (0.5)	3.8 (0.7)	4.0 (0.4)
RFQu (hypomentalizing)	0.6 (0.5)	0.6 (0.5)	0.6 (0.4)
RFQc (hypermentalizing)	1.1 (0.7)	1.1 (0.7)	1.1 (0.6)

PSS: perceived stress scale; CCQ: carer communication questionnaire; RFQu: reflective functioning questionnaire.

Multilevel analysis was also conducted on the two subscales of the RFQ. For both the hypomentalization and hypermentalization subscales, the main effect of time was not significant.

### Acceptability data (all ratings out of 10)

At session 1, the mean rating for satisfaction was 8.8 (*n* = 152, SD = 1.4) and understanding 9.2 (*n* = 151, SD = 1.2). At session 2, the mean rating for satisfaction was 9 (*n* = 128, SD = 1.1) and understanding 8.9 (*n* = 128, SD = 1.5). At session 3, the mean rating for satisfaction was 9.1 (*n* = 115, SD = 1.1) and understanding 9.2 (*n* = 117, SD = 1.1). At session 4, which is a rating of the course overall, the mean rating for satisfaction was 9.2 (*n* = 133, SD = 1.2) and understanding 9.1 (*n* = 134, SD = 1.1), and the mean rating for confidence to recommend EC to a friend was 9.5 (*n* = 125, SD = 1).

## Discussion

The results indicate that EC has a statistically significant positive impact on care partner stress levels and communication. Both outcomes improve post-intervention, and the improvements continued at follow-up. High understanding and satisfaction ratings indicate that EC is an acceptable intervention.

Two aspects of these findings are particularly notable. Firstly, stress levels decreased in spite of the fact that some care partners did not have high stress levels when they started. The course is offered to care partners who are experiencing significant stress but also to those who are not. Secondly, that the improvements on both measures continued at 4-month follow-up. This is notable as only a limited number of interventions have demonstrated ongoing improvements at follow-up (Morris et al., 2017). A randomized controlled trial (RCT) of a memory and communication training found a significant difference in knowledge of communication and other strategies over time, including 6-month follow-up (Liddle et al., 2012). In addition, a RCT of an individual coping skills intervention for care partners experiencing psychological distress found, over a 6-year follow-up period, an average improvement of two points on a measure of anxiety and depression compared to a Treatment As Usual control (Livingston et al., 2020).

To illustrate the differences between EC and other interventions that support care partners with stress and communication, we will draw out some differences between EC and an established individual coping skills intervention (STrAtegies for RelaTives, START; Livingston et al., 2013). START targets coping skills based on significant cross-sectional evidence that certain coping strategies can be more or less adaptive for carers of people living with dementia (Li et al., 2012). A meta-analytic review found that dysfunctional coping strategies, such as denial, self-criticism, avoidance and emotional discharge (letting feelings out), were moderately correlated with depression and anxiety within high-quality studies (Li et al., 2012). However, cross-sectional and longitudinal studies indicate that solution-focused coping (which is commonly associated with adaptive functioning) does not have a consistent positive impact on carer mental health (Li et al., 2012). The Communication Empowerment Framework is informed by a self-regulatory model (examples of self-regulatory models are within Carver & Scheier, 2017; Vancouver & Purl, 2017); it specifies that whether coping strategies are more or less adaptive (or dysfunctional) is determined by whether these strategies continue to serve individual goals in specific contexts (Morris & Mansell, 2018; Morris et al., 2018a). This is in line with evidence that indicates that it is the flexibility and variability in how consistently coping and other strategies are applied that influences how adaptive they are; this evidence is more consistent regarding the effect of context on adaptive coping strategies, such as problem-solving (Aldao & Nolen-Hoeksema, 2012). This emphasis on goals within the Communication Empowerment Framework and EC encourages carers to adapt how they find solutions in light of changing contexts and goals. For example, a care partner goal could be ‘maintaining verbal communication with the person they are supporting’, and a problem-solving strategy could be to focus on topics they know the person is confident speaking about. However, if the person they are supporting becomes unable to communicate verbally, they will have to modify this goal. Specifically, EC differs from START in that it draws less heavily on the coping skills literature and traditional CBT techniques (such as cognitive restructuring), and within EC, the intervention rationale is explained in terms of mentalization, control and goals rather than traditional CBT constructs (such as thoughts, feeling and behaviour cycles).

EC is designed to be an inclusive intervention aimed at care partners with a range of relationships to the person they are supporting, including spousal, child, sibling and in-law. Although it provides techniques and understandings that can help manage and reduce stress, it is not aimed purely at those experiencing psychological distress. For example, it is not delivered as a therapy or treatment through mental health services and aims at supporting communication and relationships (as well as reducing psychological distress where relevant). EC is able to target a range of needs (communication, connection, relationships and psychological health) due to its detailed underlying conceptual framework (the Communication Empowerment Framework) (Morris et al., 2018a). It draws on a detailed evidence-based theoretical and practical model of communication across dementia diagnoses, the Communicative Impact Model, which indicates that the adaptations required to respond to communication changes are similar across types of dementia diagnoses (Wray, 2020). For example, language production and comprehension may change differently across dementia types, but adaptations are more similar, such as relying on a range of communication mediums and being curious about goals and needs (Morris et al., 2018a).

The one process measure that was included within this study was of mentalization, via an adapted version of the RFQ. This was an exploratory use of this measure as it has primarily been validated as a ‘trait measure’ (i.e. not a measure of change) on individuals with severe mental health problems (e.g. borderline personality disorder) (Fonagy et al., 2016). Items were added to this measure to enhance its ability to assess the care partner’s ability to mentalize the person living with dementia. The results were not significant for either of the two subscales of this measure. As this is the first time this measure has been used in this context, it is possible that it does not measure mentalizing effectively enough within care partners or that it is not suitable as a measure of change. Whilst it is recommended by the Medical Research Council guidelines that attempts are made to evaluate the mechanisms of change within complex interventions, future studies of EC mentalization would need to be measured in a different way or one of the other key mechanisms measured instead (Craig et al., 2008; Morris et al., 2018a).

The recruitment and retention data indicated EC is a feasible intervention. Attrition levels were comparable to other interventions (e.g. Cristancho-Lacroix et al., 2015) and were substantially higher from booking to attending. Once care partners had attended, 92% attended again. The use of a facilitator manual, care partner handbook and other resources (e.g. videos), which were well-received by participants and facilitators, provides a strong basis for a replicable standardized intervention (Cramer et al., 2011).

There are some limitations to this study. The main one is that because it was not a randomized controlled trial, there are a number of sources of bias that could have influenced the results obtained. A randomized trial would be needed to reduce potentially confounding variables, such as stress improving for care partners for reasons other than accessing EC; however, given that dementia is a progressive condition, it is reasonable to conclude that stress levels would not improve without some form of intervention. There is also not an obvious reason why communication would improve as a result of something other than the EC intervention. Risk of bias through different facilitator attributes has been mitigated through manualized training of facilitators and a standardized approach to their orientation and supervision. Another limit is that the process measure (RFQ) that was used had not been tested with carers of people living with dementia, due to the lack of measures of mentalization in this context. In retrospect, it would have been better to measure one of the other key mechanisms using a measure that had been used with carers of people living with dementia. For example, the Carer Ambivalence Scale (Losada et al., 2017) measures goal conflict which is a key mechanism within the Communication Empowerment Framework (Morris et al., 2018a). A further limitation is that all the measures used were self-report. It was beyond the resources of this feasibility study to collect observational data of the interactions between care partners and people living with dementia before and after EC. However, such observational data would be useful in establishing the implementation and effectiveness of the strategies taught within EC.

Overall, this study provides evidence that EC is an acceptable and feasible intervention for care partners of people living with dementia.

## Note

1. The terms ‘family carer’, ‘care partner’ and ‘informal carer’ are often used interchangeably and refer to people undertaking unpaid caring activities, for example, a spouse or friend. Based on previous consultations with people living with dementia and carers, ‘care partners’ is used for informal carers and ‘people living with dementia’ for those they are supporting (Farina et al., 2017; Young et al., 2011).
